# The New Cottage Hospital, Axminster

**Published:** 1912-06-22

**Authors:** 


### The New Cottage Hospital, Axminster.

In November last we gave some account of this institution, which has now been transferred to the new building erected in Chard Street, and which was formally opened last Tuesday. The following is a description of the building :—

"The problem of planning the new hospital on the irregularly-shaped site of little more than a quarter of an acre presented several difficulties. The design adopted was the result of a competition, Mr. Leslie T. Moore, A.R.I.B.A., of Gray's Inn, W.C., being appointed architect for the work.

"A lofty building situated on the south-west boundary, casting its shadow over half the site after noon, and the fall of the ground, largely determined the disposition of the building, which forms two sides of a sheltered garden open to the south.

"The whole of the patients' quarters are planned on the ground floor, the two wards being arranged at an obtuse angle to one another, with a nurses' duty room between for supervision; the best orientation being attained, as far as the limits of the site would allow, with protection from the cold winds, the north point bisecting the angle formed by the wards.

"The cubic space in the wards is 1,000 feet per patient, with a floor area of 90 feet per bed. Double-hung sash windows with hopper-hung fanlight sashes over have been adopted in the wards, and the cottage casement type in the administrative portion of the building, which gives a homely appearance. The board room and matron's room flank the entrance, with the nurses' bedroom and bathroom accommodation over, forming a distinct block with a cheerful aspect. The matron's sitting-room window commands the garden. Advantage has been taken of the fall in the ground to provide a well-lighted basement under the kitchen offices for the heating chamber, coals, and washhouse, etc., by which arrangement these adjuncts are concentrated and accessible under cover. The kitchen has a north-east aspect, and is so planned that it is not a thoroughfare, both larder and pantry being accessible to the nurses. The servants' bedrooms are arranged over the kitchen block, with separate lavatory accommodation. A small operating theatre with a good bay window to the north-east and top light and ventilation is conveniently arranged.

"The building is constructed of local brick with cement rough cast, externally finished with an ochre wash. 'Shepwood' partition bricks have been adopted for several internal walls, which are finished with Sirapite plaster. All the patients' quarters have an enamel-painted dado. The roofs are covered with Bridgewater interlocking tiles, having boarding and felt under.

"The whole of the ground floor has been finished with patent non-absorbent, jointless flooring, executed by the British Doloment Flooring Company, of Westminster. The lighting is by gas. The elevations of the building are treated in a very simple manner, proportion being carefully studied and all unnecessary ornamental features omitted. The appearance of a public institution has been purposely avoided, as it was felt that a more homely type of elevation is preferable for a 'cottage' hospital. The total cost of the building was £1,750."

